# Correction: Bond breaking of furan–maleimide adducts *via* a diradical sequential mechanism under an external mechanical force

**DOI:** 10.1039/d3sc90024j

**Published:** 2023-02-15

**Authors:** Manuel Cardosa-Gutierrez, Guillaume De Bo, Anne-Sophie Duwez, Francoise Remacle

**Affiliations:** a UR Molecular Systems, Department of Chemistry, University of Liège 4000 Liège Belgium fremacle@uliege.be; b Department of Chemistry, University of Manchester Manchester M13 9PL UK

## Abstract

Correction for ‘Bond breaking of furan–maleimide adducts *via* a diradical sequential mechanism under an external mechanical force’ by Manuel Cardosa-Gutierrez, *et al.*, *Chem. Sci.*, 2023, https://doi.org/10.1039/D2SC05051J.

The authors regret that a typo in the corresponding author designation in the list of authors, appeared in the original manuscript. The corrected list of authors for this paper is as shown above.

The Royal Society of Chemistry apologises for these errors and any consequent inconvenience to authors and readers.

## Supplementary Material

